# Roles of Biomarkers in Myocardial Fibrosis

**DOI:** 10.14336/AD.2020.0604

**Published:** 2020-10-01

**Authors:** Yuejia Ding, Yuan Wang, Wanqin Zhang, Qiujin Jia, Xiaoling Wang, Yanyang Li, Shichao Lv, Junping Zhang

**Affiliations:** ^1^First Teaching Hospital of Tianjin University of Traditional Chinese Medicine, Tianjin 300193, China; ^2^Tianjin Key Laboratory of Traditional Research of TCM Prescription and Syndrome, Tianjin 300000, China; ^3^Qian’an Hospital of Traditional Chinese Medicine, Qian’an 064400, China; ^4^Tianjin Medical University Cancer Institute and Hospital, Tianjin 300060, China

**Keywords:** biomarkers, collagen, myocardial fibrosis, review

## Abstract

Myocardial fibrosis is observed in various cardiovascular diseases and plays a key role in the impairment of cardiac function. Endomyocardial biopsy, as the gold standard for the diagnosis of myocardial fibrosis, has limitations in terms of clinical application. Therefore, biomarkers have been recommended for noninvasive assessment of myocardial fibrosis. This review discusses the role of biomarkers in myocardial fibrosis from the perspective of collagen.

## 1.Introduction

Myocardial fibrosis is characterized by alterations of the extracellular matrix and is an integral part of most cardiac pathological conditions [1]. Currently, five types of collagen are known to be expressed in the myocardium. The cardiac extracellular matrix is primarily composed of fibrillar collagen type I (85%) and type III (11%) [2, 3]. Small amounts of collagen type IV and V are found in the basement membrane of myocytes and in the pericellular space [4, 5]. Besides, fibrillar collagen type VI is related to the adhesion of cellular fibers [6]. Extracellular collagen not only maintains cardiac fiber alignment, but also influences ventricle stiffness [7]. Moreover, an imbalance in collagen synthesis, breakdown, and metabolism accounts for the occurrence and development of myocardial fibrosis. Endomyocardial biopsy is undoubtedly the gold standard for the diagnosis of myocardial fibrosis, but it has limitations in clinical application. Therefore, biomarkers should be considered as a noninvasive method for detecting fibrosis. Myocardial fibrosis biomarkers have been investigated extensively, and fibrosis markers play an important role in the prognosis of cardiovascular diseases, such as heart failure (HF), dilated cardiomyopathy (DCM), and hypertrophic cardiomyopathy (HCM). This review summarizes the biomarkers of myocardial fibrosis from four collagen-related perspectives: synthesis, breakdown, metabolism, and gene transcription (miRNA).

## 2.Collagen Synthesis

### 2.1 C-Terminal Propeptide of Procollagen Type I

Type I collagen is a heterotrimeric molecule, which is composed of two α 1 chains and one α 2 chain. During its synthesis, the protein undergoes a series of post-translational modifications to form the procollagen chain; this precursor is then secreted into the extracellular space and cleaved by specific proteinases [8]. The stoichiometric ratio of the C-terminal propeptide of procollagen type I (PICP), which is released into the blood, and collagen type I produced by cleavage is 1:1 [9]. The heart secretes PICP into the peripheral circulation through the coronary sinus [9]. However, whether there is a positive correlation between plasma PICP, and myocardial collagen content remains controversial. A cross-sectional study demonstrated that plasma PICP levels in HCM patients were positively correlated with myocardial PICP content and the histological myocardial collagen volume fraction [10]. Similarly, Ferreira et al. also confirmed that the serum levels of PICP were significantly higher in patients with hypertension before drug treatment [11]. In contrast, in heart failure rats, Adamcova et al. observed that collagen content in the left ventricle was increased, whereas plasma PICP levels were reduced by 42% as compared to the control group [12]. These findings suggest that the myocardial collagen content does not necessarily correlate with plasma PICP, which may be related to confounding factors (such as weight loss and catabolic state). For predictive value, Ruiz-Ruiz et al. considered that serum PICP can be used independently for predicting HF episodes, hospital readmission, and death [13]. They included 111 patients with decompensated HF and found that 22.52% of the patients died during the 21 months of follow-up, and 48.6% of the patients were readmitted for HF. Moreover, serum PICP levels were significantly increased among patients who reached some primary endpoints (new hospitalizations or death) during follow-up (88.12 ± 37.31 ng/mL vs 73.13 ± 34.06 ng/mL; p = 0.029), and a cut-off value of 124 ng/mL predicted prognosis most accurately. Unfortunately, they did not perform cardiac biopsies, which is considered the most reliable method for measuring myocardial fibrosis.

### 2.2Procollagen Type I N-Terminal Propeptide

Excessive deposition of collagen type I is a feature of cardiac remodeling [14]. The procollagen type I N-terminal propeptide (PINP) originates from conversion of collagen type I, and is a marker for collagen type I synthesis [15]. N-terminal pro-peptides are removed by members of the ADAMTS family [16]. Compared with PICP, PINP has the disadvantage of delayed release [17]. At present, the relationship between PINP levels and fibrosis remains elusive. In a case?control study, Zile et al. observed that the baseline serum PINP levels in patients with HF were significantly higher than those in controls [18]. In rats with ischemic cardiomyopathy, Zhang et al. detected significant cardiac fibrosis by Masson staining 6 weeks after myocardial infarction (MI), and enzyme-linked immunosorbent assay (ELISA) results showed that the level of plasma PINP in the MI group was significantly increased as compared to that in the normal control group [19]. However, in a cross-sectional study in which serum PINP levels were measured by radioimmunoassay, no significant difference was revealed between HCM patients and healthy individuals [20]. As shown by the above studies, PINP has some limitations as a biomarker.

### 2.3. Procollagen Type III Amino-Terminal Propeptide

Collagen type III, whose fibers have a relatively small diameter, is synthesized by cardiac fibroblasts and is mainly responsible for myocardial elasticity [21, 22]. After the C-propeptides of fibrillar procollagen are cleaved by proteases, procollagen type III amino-terminal propeptide (PIIINP) is released into the blood via the lymphatics [17]. The N-propeptide is considered to play an important role in the regulation of primary fibril diameter [23]. Excellent storage and stability are the characteristics of PIIINP [24]. However, PIIINP is sometimes not completely separated from procollagen, which leads to the underestimation of type III collagen synthesis. In a prospective study of dilated cardiomyopathy, baseline serum PIIINP levels in patients with idiopathic or ischemic dilated cardiomyopathy were significantly higher than those in healthy controls, and serum PIIINP levels were highly correlated with cardiac collagen type III levels [25]. Patients with a serum PIIINP value > 7 pg/L had a higher risk of a poor hemodynamic condition, advanced clinical stage, heart transplantation, hyponatremia, and death during follow-up than did patients with low PIIINP values [25]. This shows that the increase in serum PIIINP levels could reflect cardiac fibrosis to some extent, which was of prognostic value and was related to clinical stage. Additionally, PIIINP levels were positively correlated with diastolic dysfunction in patients with HF with reduced ejection fraction (HFrEF) [26], with left ventricular mass index (LVMI), and with relative wall thickness (RWT) in patients with successfully repaired coarctation of the aorta (CoA) with left ventricular hypertrophy [27]. It was also inversely associated with diastolic function in patients with hypertension [28]. Moreover, in myocardial infarction (MI) model rats, the reduction in fibrosis with tanshinone IIA was accompanied by a reduction in serum PIIINP [29]. Taken together, PIIINP can reflect the degree of myocardial fibrosis.

## 3.Collagen Breakdown

### 3.1 C-Terminal Telopeptide of Collagen Type I

During degradation of collagen type I fibrils, the C-terminal telopeptide of collagen type I (CITP) is a cross-linked terminal peptide released in a 1:1 stoichiometric ratio, allowing accurate measurement of collagen degradation [30, 31]. In a cross-sectional study, serum CITP levels in HCM patients were increased, while the PICP and PINP levels were not altered significantly, indicating a shift in the collagen balance toward collagen type I breakdown [20]. This is an important outcome, because increased myocardial stiffness is usually caused by collagen deposition. However, several other studies have shown that the relationship between CITP and myocardial fibrosis is controversial. In patients with HF and atrial fibrillation (AF), serum CITP levels were significantly higher than those in the control groups [32, 33]. But Nagao et al. observed that serum CITP levels in patients with DCM were not associated with left ventricular remodeling parameters, or with the expression of cardiac collagen type I and type III [34]. In addition, some studies have confirmed the predictive value of CITP. Manhenke et al. considered that plasma CITP was an independent predictor of cardiovascular mortality in patients with acute myocardial infarction (AMI) [35]. This prospective study included 233 patients with AMI, among whom 56% reached the combined endpoint of HF symptoms or CV death during the years of follow-up, and plasma ICTP was increased in patients who died due to any cause. Similarly, serum CITP is also useful for determining cardiac events in patients with complete HF [36]. In summary, CITP may facilitate diagnosis or prognosis of myocardial fibrosis.

### 3.2 Matrix metalloproteinases

Matrix metalloproteinases (MMPs) are zinc- and calcium-dependent peptide enzymes that are involved in myocardial remodeling and degradation of collagen [37, 38]. It promotes the degradation of extracellular matrix, such as collagen, elastin, and gelatin [39]. MMPs can hydrolyze collagen efficiently, but their relative activities towards interstitial collagens are different. At 25°C, MMP-1 preferentially cleaves collagen type III over types I and II [40]. MMP-13 cleaves collagen type II five-fold faster than collagen type I and six-fold more rapidly than collagen type III, at 25 °C [41]. For MMP-2 and MMP-9, collagen type III is the substrate preferred over collagen types I and II [42, 43].

Many MMPs are expressed in the myocardium. In HCM, the expression of MMPs is altered, but not all MMPs are affected. Münch et al. found that MMP-1, MMP-2, MMP-3, and MMP-9 are involved in HCM [44]. They observed that increased serum MMP-2 levels in females were associated with lower fibrosis, while MMP-9 was positively associated with fibrosis in late gadolinium enhancement cardiac magnetic resonance (mean increase of 0.66 g/unit MMP-9 [0.50;0.82], p < 0.001), but neither serum MMP-1 nor MMP-3 levels were associated with cardiac fibrosis [44]. However, a cautionary note on these results is that the cardiac tissue concentration of MMPs was not evaluated, which might present a limitation. Regarding MMP-1 levels in systolic and diastolic HF, serum MMP-1 levels were increased in systolic HF patients, but not in diastolic HF patients [45, 46]. This result reminds us that MMP activity also fluctuates because heart remodeling during HF is a dynamic process. In diabetic mice (DM), myocardial fibrosis is clearly related to the expression of MMP7, MMP11, MMP13, and MMP16 in myocardial tissue [47]. Compared with the control group, levels of MMP7, MMP11, MMP13, and MMP16 were markedly higher, but levels of MMP2 were markedly lower in the DM group [47]. It is also important to note the measurement method of MMPs in all the above studies. The levels of different MMPs are determined by immunohistochemistry or histology or by enzyme-linked immunosorbent assays, but these methods do not distinguish proMMPs from active MMPs. Therefore, these results are of limited value.

### 3.3 Tissue Inhibitors of Metalloproteinase

Tissue inhibitors of metalloproteinase (TIMPs) are a group of low molecular weight glycoproteins. TIMPs are produced and secreted by fibroblasts, epithelial cells, and endothelial cells, and are distributed among tissues and humors [48]. TIMPs can promote the differentiation of fibroblasts into myofibroblasts at the site of tissue injury [49]. In addition, TIMPs control the proteolytic activity of MMPs [50]. Specifically, TIMPs can specifically bind to the zinc ions in the catalytic site of MMPs through the cysteine residues, thus disbanding activated MMPs, or preventing inactive MMPs from becoming activated [47]. Therefore, the balance between MMP and TIMP expression is essential for reconstruction of the extracellular matrix in myocardial tissues [51].

Currently, 4 types of TIMPs have been found to be associated with myocardial fibrosis. TIMP1 mediates the interaction between fibroblast membrane protein CD63 and integrin β1, which is widely expressed in various cells, thus triggering fibrosis [52-55]. At the cellular level, hirudin reverses Ang II-induced fibrosis by elevating TIMP-2 expression [56]. TIMP3 is the only TIMP that can inhibit the activity of TNF-α-converting enzyme and may have a protective effect against fibrosis by upregulating cytokines involved in myofibroblast activation and immunity [57]. In addition, Rizzi et al. investigated whether there was a correlation between cardiac TIMP-4 levels and cardiac hypertrophy in 2K1C hypertension rats [58]. They observed higher TIMP-4 levels (325 ± 68%) in hypertensive rats than in sham-operated animals at 75 days after 2K1C surgery. This result suggested that the increase in TIMP-4 activity was associated with concomitant development of cardiac hypertrophy. Another way to determine the severity of fibrosis is by measuring the MMP/TIMP ratio. Parkkonen et al. observed a large increase in serum TIMP-1 and MMP-2 levels in patients with takotsubo cardiomyopathy (TTC) compared with that in healthy controls, and proposed that the low MMP-8/TIMP-1 molar ratio may reflect increased transient fibrosis and decreased proteolysis [59]. This result indicates that the TIMP/MMP balance may play an important role in the development of myocardial fibrosis. In addition, the predicted value of TIMP-1 is also noteworthy. Frantz et al. investigated the association between TIMP-1 plasma levels and clinical endpoints (death due to any cause), and found that plasma TIMP-1 levels increased in HF patients as compared with healthy controls (1640 vs 735 ng/mL, respectively,) [60]. Moreover, the researchers also observed that patients with high TIMP-1 levels (1917 ng/mL) had a significantly higher mortality rate than patients with lower levels (1390 ng/mL). These results suggest that an increase in TIMP-1 indicates a poor prognosis in HF patients.

**Figure 1.**
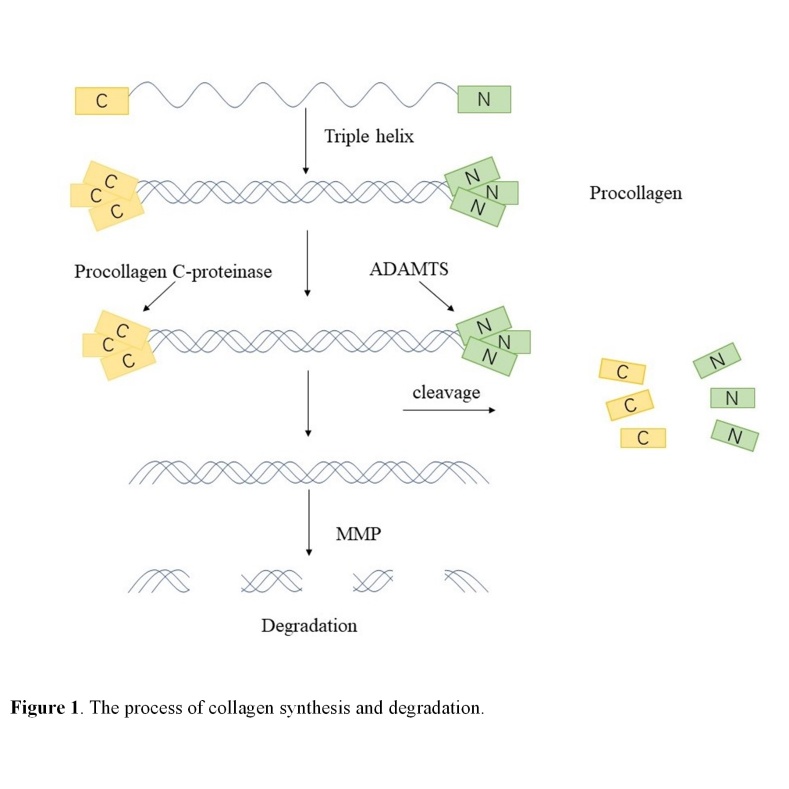
The process of collagen synthesis and degradation.

The collagen synthesis and degradation processes are briefly described in Fig. 1.

## 4.Collagen Metabolism

### 4.1 Transforming Growth Factor-β and Smads

Transforming growth factor-β (TGF-β) is a crucial profibrotic cytokine involved in myocardial fibrosis [61]. It is involved in the regulation of fibroblast proliferation, transformation, and migration, and production of the extracellular matrix [62]. As a cytokine upstream of LOX in cardiac fibroblasts, TGF-β activates fibrosis via the Smad3, PI3K/Akt, and MAPK signaling pathways [63]. In addition, the heterodimeric cell surface receptor complex composed of type I TGF-β receptors and type II TGF-β receptors plays an important role in signal transduction of cardiac fibroblasts [64, 65].

In various animal models of heart disease, inhibition of the effect of TGF-β successfully reduced or prevented the development of fibrosis. In MI mice, anti-TGF-β treatment after coronary artery ligation increased the expression of MMPs and decreased the production of collagen [66]. Similarly, in pressure-overloaded rats, the delivery of anti-TGF-β neutralizing antibody inhibited the activation of fibroblasts and prevented the induction of collagen type I and type III mRNA [67]. These observations indicated that anti-TGF treatment prevented excessive extracellular matrix deposition. Moreover, Koitabashi et al. observed that deletion of the TGF-β type II receptor (TβR2) gene in cardiomyocytes inhibited cardiac hypertrophy, and that transverse aortic constriction (TAC) mice receiving anti-TGF agents displayed significantly suppressed perivascular and interstitial fibrosis [68]. Taken together, these data emphasize the key role of TGF-β in cardiac fibrosis.

Smads, as key intracellular effectors of the TGF-β1 class, and play a central role in cardiac remodeling [69, 70]. Research has suggested that the up-regulatory effect of TGF-β3 on the migration, proliferation, and collagen synthesis of human cardiac fibroblasts may be attributed to the action of Smad7 [71]. Khalil et al. found that the absence of Smad3, Smad2/3, or Tgfbr1/2 markedly attenuated cardiac fibrosis after 12 weeks of TAC stimulation in rats [72]. In Smad3-deficient mice, the collagen content in the infarcted hearts decreased at 7 days after reperfusion, and collagen deposition in the uninfarcted, remodeling myocardium was markedly attenuated [73]. These findings suggest that Smad gene disruption leads to attenuation of the remodeling process.

### 4.2 Connective Tissue Growth Factor

Connective tissue growth factor (CTGF) belongs to the CCN family of multifunctional matricellular proteins, and it is involved in the process of triggering fibrosis in multiple organs and tissues, including the heart [74, 75]. Many in vitro studies have demonstrated that CTGF promotes the differentiation of fibroblasts into myofibroblasts and enhances extracellular matrix production [76-78]. CTGF is also a critical mediator of the downstream signaling of the profibrotic cytokine TGF-β [79, 80]. In DCM model rats, CTGF/CCN2 gene silencing improved cardiac function, attenuated myocardial fibrosis, and left ventricular hypertrophy [81]. In an in vitro study, β-adrenergic receptor overstimulation could induce the synthesis and secretion of CTGF in cardiomyocytes, thereby affecting the activation of cardiac fibroblasts [82]. Dean et al. observed the expression of spatiotemporal CTGF during the development of myocardial fibrosis in experimental MI rats [83]. They confirmed that the expression of CTGF protein and mRNA was upregulated rapidly in the infarcted region within a few days after MI [83]. These findings suggest that CTGF plays a key role in myocardial fibrosis. Furthermore, Koitabashi et al. proposed that plasma CTGF could be used as a diagnostic marker for chronic HF [84]. They found that plasma CTGF levels in symptomatic choric HF patients were significantly increased in proportion to their NYHA classes and were correlated with plasma BNP concentration (r = 0.395, P<0.01). However, they did not measure the concentration of CTGF in aortic root blood and coronary sinus blood; thus, the source of CTGF production is unknown. In brief, CTGF may be a predictor of myocardial fibrosis.

### 4.3 Corin

Corin is a transmembrane protease that is mainly expressed in cardiomyocytes [85]. It is closely correlated with natriuretic peptides that regulate many signaling functions, such as cGMP levels, vasodilation, natriuresis, fibrosis, etc. [86, 87]. In DCM model mice, increasing the expression of cardiac corin not only reduced fibrosis and HF, but also increased survival [88]. This finding indicates that corin correlates with the prognosis of DCM. In a cross-sectional study, plasma corin levels were lower in HF patients (365 pg/mL [±SD, 259 pg/mL]; P < 0.001) than in healthy controls, and this reduction was closely related to the severity of the disease (P < 0.001 for NYHA class II vs. class IV; P < 0.05 for NYHA class III vs. class IV) [85]. The results demonstrated that plasma corin may indicate pathological conditions in the heart. In canines with HF, the staining intensity of collagen was markedly elevated, while the expression of corin mRNA and protein was lower than that in the control group [89]. Contrary to these findings, Tran et al. observed upregulation of corin gene expression in the failing myocardium and in hypertrophic cardiomyocytes [90]. These studies suggest that the expression of corin may differ according to the disease state. In summary, corin may be an attractive diagnostic and prognostic biomarker of myocardial fibrosis.

### 4.4 Mesenchymal cell products

Recently, endothelial-to-mesenchymal transition (EndoMT) has been considered as a potential mechanism in pathological fibrosis. In the complex biological process of EndoMT, endothelial cells lose their specific cellular markers, and then acquire a mesenchymal or myofibroblast phenotype to initiate expression of mesenchymal cell products, such as α-smooth muscle actin (α-SMA), vimentin, and fibronectin [91]. In a rat model of isoproterenol (ISO) -induced myocardial fibrosis, cardiac α-SMA and vimentin levels increased, peaking on day 3, and then gradually decreased [92]. This result suggests that α-SMA and vimentin activity fluctuates in the fibrosis process. α-SMA, fibronectin, and vimentin are also involved in the process of diabetic cardiomyopathy [93]. The immunohistochemistry data showed that diabetes enhanced the expression of cardiac α-SMA, fibronectin, and vimentin compared with normal rats. Moreover, in vitro treatment of mouse embryonic fibroblasts with WF-A reduced the stability of collagen mRNA, but in the absence of vimentin, WF-A did not change the half-life of collagen mRNAs [94]. We therefore conclude that vimentin filaments play a crucial role in collagen expression. In short, α-SMA, vimentin, and fibronectin might be essential elements in cardiac fibrosis.

### 4.5 Inflammatory Factors

Inflammation caused by infection and tissue necrosis is a physiological response that promotes tissue healing through fibrosis, but it may become excessive when accompanied by additional factors, such as mechanical stress, genetic background, activation of neurohumoral factors, oxidative stress, and autoimmunity, causing pathological remodeling through Gal-3, TNF-α, interleukin, MMPs, miRNA activation, and other mechanisms.

Galectin-3 (Gal-3), a β-galactoside-binding lectin, plays a regulatory role in fibrogenesis. It is widely expressed by various types of inflammatory cells, binds to extracellular glycoproteins, and activates fibroblasts to increase collagen I deposition [95-97]. Growing evidence indicates that high levels of circulating Gal-3 are associated with an increased risk of adverse cardiovascular events, such as HF, myocardial infarction, dilated cardiomyopathy, and fibrogenesis [98-101]. In mice with ischemia/reperfusion (I/R) injury, there was a two-fold increase in plasma Gal-3 levels, and in the ischemic myocardium, Gal-3 was upregulated seven-fold at the mRNA level and 30-fold at the protein level [102]. In addition, that study revealed that plasma Gal-3 concentrations were always higher in DCM/ICM patients with HF than in non-HF subjects, but no trans-cardiac or trans-hepatic concentration gradient of Gal-3 was found. The aforementioned studies were limited in that they did not obtain myocardial biopsies to measure cardiac Gal-3 expression. Moreover, serum Gal-3 is considered to be associated with inflammation and cardiovascular fibrosis. In patients with AMI, serum Gal-3 levels increased immediately after AMI and then declined significantly within 5 days [103]. These data indicate that Gal-3 is closely related to the formation, destabilization, and rupture of plaque. Besides observational studies of Gal-3 levels, several studies have investigated the predictive value of Gal-3. Van Kimmenade et al. observed that an increased circulating Gal-3 level was the best independent predictor of 60-day mortality (odds ratio 10.3, P < 0.01) in patients with acute HF [104]. In patients with coronary artery disease (CAD), Maiolino et al. found that patients with Gal-3 levels in the highest tertile were more prone to death due to cardiovascular causes (25.2%) than those with Gal-3 levels in the intermediate and lower tertiles (13.6% and 7.5%, respectively; P < 0.001), and the plasma Gal-3 cutoff value for cardiovascular death prediction was 27.7 ng/mL [105]. Unfortunately, the loss of 25% of the patients to follow-up may have limited the results of the study. Overall, these findings confirm that elevated Gal-3 is associated with cardiovascular disease progression and poor outcome.

A growing body of evidence has demonstrated that macrophages and monocytes not only play a key role in the occurrence and development of fibrotic responses, but may also mediate the regression of fibrosis [106]. Macrophages and monocytes are capable of producing and excreting a large number of pro-inflammatory mediators, such as tumor necrosis factor-α (TNF-α), interleukin-1β (IL-1β), IL-4, and IL-6 [107]. Furthermore, a complex regulatory network consisting of these inflammatory factors activates both myofibroblasts and fibrotic pathological processes [107]. This was supported by Kang et al., who treated primary cardiomyocytes with 35 mmol/L glucose for 24 h [108]. The ELISA results showed that the levels of TNF-α, IL-6, and IL-1β and the expression of collagen I and III mRNA were higher than those in the normal control group [108]. These findings suggest that fibrosis is associated with inflammation. In a cross-sectional study of HCM, Fang et al. observed that plasma IL-4, IL-6, and IL-10 levels were positively correlated with diffuse and regional myocardial fibrosis [97]. Unfortunately, owing to the cross-sectional nature of the research, it is impossible to determine whether this association was causal. In addition, in order to study the role of IL-11 signal transduction in cardiovascular fibrosis, Schafer et al. generated IL-11 knockout mice. Compared with wild-type mice, they observed less cardiac fibrosis in knockout mice after either transverse aortic constriction or AngII infusion, suggesting that IL11 is a crucial profibrotic gene [109]. In summary, inflammatory factors are involved in the pathological process of myocardial fibrosis.

**Table 1:** Biomarkers of myocardial fibrosis (collagen synthesis, breakdown, metabolism).

Biomarkers	Studied Condition	Test Location	Main findings	Reference
PICP	HCM patients	Plasma	PICP ↑	[10]
	Hypertension patients	Serum	PICP ↑	[11]
	HF rats	Plasma	PICP ↓	[12]
	HF patients	Serum	PICP ↑	[13]
PINP	HFrEF patients	Serum	PINP ↑	[18]
	MI rats	Plasma	PINP ↑	[19]
	HCM patients	Serum	Not predictive in fibrosis	[20]
PIIINP	DCM patients	Serum	PIIINP ↑	[25]
	HFrEF patients	Serum	PIIINP ↑, positively associated with diastolic function	[26]
	CoA patients	Serum	PIIINP ↑, positively associated with LVMI and RWT	[27]
	Hypertension patients	Serum	PIIINP ↑, inversely associated with diastolic functions	[28]
	MI rats	Serum	PIIINP ↑	[29]
CITP	HF patients	Serum	CITP ↑	[20]
	HF and AF patients	Serum	CITP ↑	[32-33]
	DCM patients	Serum	Not predictive in fibrosis	[34]
	AMI patients	Serum	CITP ↑, predicts cardiovascular mortality	[35]
MMPs	HCM patients	Serum	MMP-2 ↓, MMP-9 was positively associated with fibrosis	[44]
	systolic HF patients	Serum	MMP-1↑	[45-46]
	DM rats	Myocardial tissues	MMP2↓, MMP7, MMP11, MMP13, MMP16↑	[47]
TIMPs	2K1C hypertension rats	Myocardial tissues	TIMP-4↑	[58]
	TTC patients	Serum	TIMP-1 and MMP-2 ↑	[59]
	HF patients	Serum	TIMP-1↑	[60]
TGF-β	MI mice	Myocardial tissues	anti-TGF-β treated: collagen production ↓, matrix-metalloproteinase ↑	[66]
	Pressure-overload rats	Myocardial tissues	anti-TGF-β treated: fibroblast activation ↓, collagen mRNA induction ↓	[67]
	TAC mice	Myocardial tissues	anti-TGF-βtreated: perivascular and interstitial fibrosis ↓	[68]
Smads	TAC mice	Cardiac tissues	Smad3 or Smad2/3 deletion: cardiac fibrosis↓	[72]
	Smad3-deficient mice	Myocardial tissues	Collagen content and deposition ↓	[73]
CTGF	DCM rats	Myocardial tissues	CTGF/CCN2 gene silencing: cardiac function ↑, myocardial fibrosis and left ventricular hypertrophy ↓	[81]
	MI rats	Myocardial tissues	CTGF ↑	[83]
	Chronic HF patients	Plasma	CTGF ↑	[84]
Corin	HF patients	Plasma	Corin ↓	[85]
	HF canines	Myocardial tissues	Corin ↓	[89]
	HCM disease	Cardiomyocytes	Corin↑	[90]
EndoMT	ISO-induced fibrosis	Myocardial tissues	α-SMA and vimentin: ↑ with a peak on day 3, and then gradually↓	[92]
	DCM rats	Myocardial tissues	a-SMA, fibronectin and vimentin↑	[93]
	In vitro	Mouse embryonic fibroblasts	Vimentin deletion: WF-A did not change half-lives of collagen mRNAs	[94]
Gal-3	Myocardial I/R injury rats	Myocardial tissues	Gal-3 ↑	[102]
	AMI patients	Serum	Gal-3 ↑ after AMI and then ↓ within 5 days	[103]
	Acute HF patients	Serum	Gal-3 ↑	[104]
	CAD patients	Plasma	Gal-3 is positively correlated with cardiovascular deaths	[105]
TNF-α and interleukin	Cardiomyocyte fibrosis	Cardiomyocytes	TNF-α, IL-6 IL-1β levels and collagen I and III mRNA expressions ↑	[108]
	HCM patients	Plasma	IL-4, IL-6, and IL-10 ↑	[97]
	IL-11 knockout mice	Myocardial tissues	Fibrosis↓ after either transverse aortic constriction or AngII infusion	[109]
Other Molecules	HFrEF patients	Serum	RLN1↑	[115]
	ISO-induced cardiac fibrosis	Myocardial tissues	GSHR deletion: myocardial fibrosis ↑	[116]
	VHD mice	Myocardial tissues	ADAMTS-1↑	[117]

Abbreviations: PICP, C-terminal Propeptide of Procollagen Type I; PINP, Procollagen Type I N-terminal Propeptide; PIIINP, Procollagen Type III Amino-terminal Propeptide; CITP, C-terminal Telopeptide of Collagen Type I; MMPs, Matrix metalloproteinases; TIMPs, tissue inhibitors of metalloproteinase; TGF-β, Transforming growth factor-β; CTGF, connective tissue growth factor; EndoMT, endothelial to mesenchymal transition; Gal-3, Galectin-3 protein; TNF-α, tumor necrosis factor -α; HCM, hypertrophic cardiomyopathy; HF, heart failure; HFrEF, heart failure and reduced ejection fraction; MI, myocardial infarction; DCM, dilated cardiomyopathy; CoA, coarctation of the aorta; AF, atrial fibrillation; AMI, acute myocardial infarction; DM, diabetic mice; 2K1C:two-kidney one-clip; TTC, takotsubo cardiomyopathy; TAC, transverse aortic constriction; I/R, ischemia/reperfusion; ISO, isoproterenol; CAD, coronary artery disease; VHD, viral heart disease; LVMI, left ventricular mass index; RWT, relative wall thickness; RLN1, relaxin-1; GSHR, growth hormone secretagogue receptor

MMPs and miRNAs also play a regulatory role in the process of inflammatory fibrosis. Tissue injury induces activation of inflammatory mediators and recruitment of inflammatory cells. Inflammatory cells promote fibrosis by producing cytokines/chemokines that regulate MMPs [110]. MMP-9, the most widely documented protease in the inflammatory process, is involved in the initial stage of myocardial injury. The increase in MMP-9 levels is related to the invasion of necrotic tissue by inflammatory cells, particularly polymorphonuclear neutrophils and activated satellite cells [111]. Moreover, in the cardiovascular system, miRNAs control the expression of some inflammatory factors. For example, miR-155 controls the expression of SHIP1 and SOCS1, the key regulators of the inflammatory response in macrophages [112]. Elevated miR-21 expression in macrophages inhibited the production of TNF-α and the up-regulation of IL-10, respectively [113]. Overall, these inflammatory markers are associated with fibrosis.

### 3.6Other Molecules

The development of myocardial fibrosis also involves factors such as relaxin-1 (RLN1), growth hormone secretagogue receptor (GSHR), ADAMTS-1, and transient receptor potential melastatin 7 (TRPM7). RLN, a polypeptide hormone, is secreted by cardiac cells [114]. In a cross-sectional study, the average circulating RLN1 levels in the HFrEF patient population were markedly higher than those in healthy control subjects (702 ± 283 pg/mL vs. 44 ± 27 pg/mL), with elevated RLN1 levels accompanied by a decrease in heart fibrosis [115]. This finding promotes the study of RLN1 as a potential anti-fibrosis target. GSHR is an orexigenic hormone with newly defined cardiovascular effects. In a mouse model of isoproterenol-induced myocardial fibrosis, GHSR deficiency exacerbated the expression of myofibroblast trans-differentiation marker genes, suggesting that GHSR may be a surrogate indicator of the need for intervention in myocardial fibrosis [116]. ADAMTS-1, a metalloprotease with proteolytic activity, also has the ability to cleave the N-terminal propeptide of collagen. In mice with viral heart disease (VHD), Li et al. observed that the collagen volume fraction increased significantly, accompanied by elevated expression of ADAMTS-1 mRNA in cardiac tissue (P < 0.001) [117]. Moreover, TRPM7 is capable of promoting the proliferation and differentiation of fibroblasts and increasing the synthesis of extracellular matrix proteins [118].

In general, these molecules, which are closely related to collagen synthesis, breakdown, and metabolism, have recently been proposed as intervention targets for myocardial fibrosis (Table 1).

## 5.Gene Transcription (miRNA)

MicroRNAs (miRNAs) are small, noncoding RNAs that regulate gene expression after transcription, and play a critical role in heart function and pathology [119]. The miRNA family regulates gene expression by inducing target mRNA destabilization or inhibiting protein translation [120]. The marked cardiospecific capacity of some miRNAs is beneficial for ameliorating tissue remodeling.

The relationship between miR-21 and myocardial fibrosis has been extensively studied. miRNAs target not only single genes, but also overall networks that contribute to biological function. For example, in patients with aortic stenosis (AS), myocardial and plasmatic miR-21 levels predicted collagen type I, collagen type III, and fibronectin expression by targeting RECK, PDCD4, and TGF-β-signaling factors [121]. In animal models of myocardial infarction, miR-21 promoted the transformation of cardiac fibroblasts (CFs) to myofibroblasts, and increased myocardial fibrosis in vivo by targeting Jagged1 [122]. In addition, the overexpression of miR-21 in allogeneic mice activated the fibrosis gene program and promoted the differentiation of monocytes to fibroblasts via the phosphatase and tensin homologue/activator protein 1 regulatory axis (PTEN/AP-1) [123]. Intriguingly, Szemraj-Rogucka et al., in a study of 13 left ventricular non-compaction patients (LVNC), found that plasma levels of miR-21, miR-29a, miR-30d, and miR-133a were both significantly elevated, suggesting that all four miRNAs may serve as biomarkers of myocardial fibrosis [124]. Unfortunately, this study was performed using a small sample size, which may have biased the results.

**Table 2:** Biomarkers of myocardial fibrosis (gene transcription).

MiRNA	Study condition	Pathway	Main findings	Reference
miR-21	AS patients	RECK,PDCD4, and TGF-β	Predicts myocardial collagen expression	[121]
	MI mice	Jagged1	CFs?myofibroblasts transformation↑, myocardial fibrosis ↑	[122]
	Cardiac allograft transplantation model	PTEN/AP-1 pathway	Fibrosis gene program ↑, monocytes-fibroblasts transformation ↑	[123]
	LVNC paitients	—	Plasma miR-21, miR-29a, miR-30d and miR-133a↑	[124]
miR-29	Pathological hypertrophy model	—	Cardiac miR-29a, mir-29c ↓	[127]
	Cardiac hypertrophy mice	—	Prevented Col1a1, Col1a2 and Col3a1 expression	[128]
miR-1	Myocardial hypertrophy model	FBLN2	Cardiac remodeling ↓	[129]
miR-378	TAC rats	Paracrine mechanisms	Cardiac fibrosis ↓	[130]
miR-203	DCM rats	PI3K/Akt signaling pathway	Prevented cardiac Col I, Col III expression	[131]
miR-135a	Cardiac hypertrophy model	TGF-β/Smads pathway	Associated with the α-SMA and Co I	[132]
miR-135a, miR-202-3p, miR-122, miR-195 and miR-328	Fibrosis model	TGFβ1 signaling pathway	Involved in the progression and development of myocardial fibrosis	[132-136]
miR-197-5P	HF patients	—	miR-197-5P↑, associated with adverse cardiac events	[138]
miR-208, miR-499	AMI paitients	—	Mediates cardioblasts?cardiomyocytes transformation and muscle fiber specification	[139]
miR-101, miR-150	MI rats	—	Cardiac miR-101 and miR-150 ↓	[140-141]
miR-144	MI model	—	miR-144 deletion: cardiac collagen content ↑, cardiac function ↓	[142]
miR-101a	MI rats	—	Intermittent aerobic exercise: cardiac miR-101a ↑	[143]

Abbreviations: AS, aortic stenosis; MI, myocardial infarction; LVNC, left-ventricular non-compaction; TAC, transverse aortic constriction; DCM, dilated cardiomyopathy; I/R, ischemia/reperfusion; HF, heart failure; AMI, acute myocardial infarction; CFs, cardiac fibroblasts; FBLN2, Fibullin-2; PTEN/AP-1, phosphatase and tensin homologue/activator protein 1 regulatory; α-SMA, α-smooth muscle actin; TGF-β, Transforming growth factor-β

**Figure 2.**
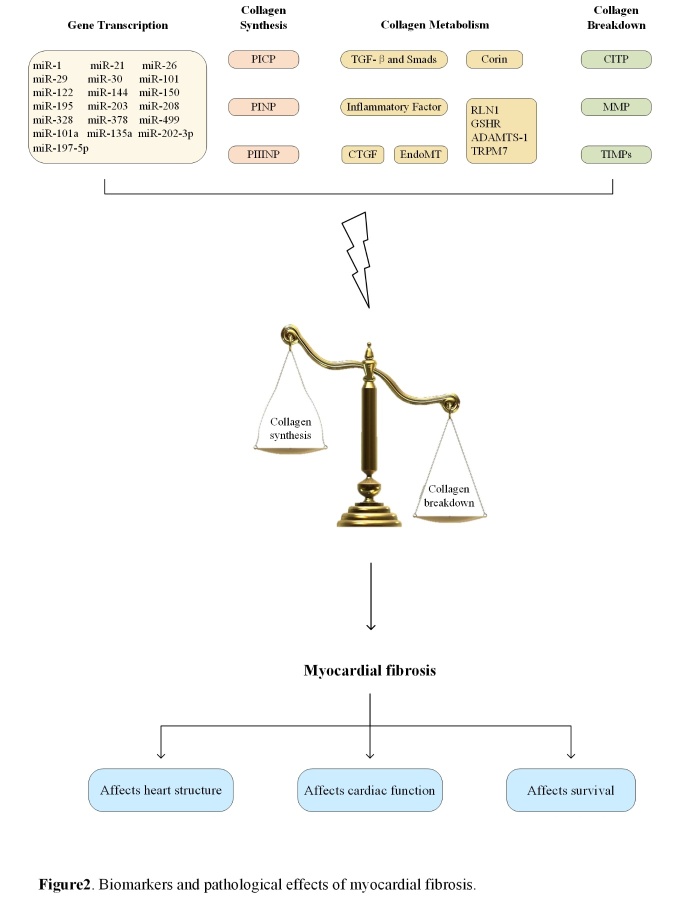
Biomarkers and pathological effects of myocardial fibrosis.

Another well-researched miRNA is miR-29, which demonstrates reduced expression under cardiac stress conditions, and thus may promote more extracellular matrix protein production by “derepression” of elastin, genes encoding collagens, and fibrillin [125]. Heid et al. found that overexpression of the miR-29 family counteracted the physiological accumulation of oxidative damage during aging, thereby protecting the heart against the detrimental effects of fibrosis [126]. In patients with pathological hypertrophy, TaqMan quantitative polymerase chain reaction showed a marked decrease in cardiac miR-29a and miR-29c, suggesting that miR-29 expression has been linked to extracellular matrix remodeling in cardiac hypertrophy [127]. MiR-29 is also a good example of a potential therapeutic target. For example, infusion of anti-miR-29 into cardiac hypertrophy model mice could prevent the expression of fibrosis markers, such as Col1a1, Col1a2, and Col3a1 [128]. Together, the miR-29 family plays a key role in pathological hypertrophy of the myocardium and fibrosis.

Other cardiomyocyte-associated miRNAs, such as miR-26, miR-30, miR-135, and miR-208, are also involved in cardiac fibrosis. miR-1 expression reverses pressure-induced myocardial hypertrophy and prevents cardiac remodeling by secretion of the protein fibullin-2 (FBLN2), which is related to extracellular matrix remodeling [129]. miR-378 is secreted from cardiomyocytes at the early stage of cardiac remodeling after mechanical stress and inhibits excessive cardiac fibrosis through paracrine mechanisms [130]. Overexpression of miR-203 prevented cardiac collagen type I and type III expression in DCM mice by targeting PIK3CA through inactivation of the PI3K/Akt signaling pathway [131]. As upstream molecules of TGF-β, miR-135a, miR-122, miR-195, miR-202-3p, and miR-328 also participate in the process of fibrosis by regulating the expression of myocardial collagen [132-136]. Rubiś et al. found that baseline serum levels of miR-21, miR-26, miR-29, and miR-30 were significantly different in DCM patients than in controls, and that MMP-2 levels were strongly associated with all of the microRNAs studied [137]. In a prospective study, elevated baseline plasma miR-197-5P levels were considered to be associated with myocardial fibrosis in patients with end-stage HF, and during the average 937-day follow-up period, 22 patients (27.5%) had major adverse cardiac events, including 19 deaths and three cardiac transplantations [138]. Additionally, multiple studies have found evidence implicating cardiac interstitial fibrosis in the deterioration of cardiac function in myocardial infarction. In AMI, miR-208 and miR-499 mediate cardioblast?cardiomyocyte transformation and fast/slow muscle fiber specification at the late cardiogenic stages [139]. In addition, 4 weeks after coronary artery ligation in rats, miR-101 and miR-150 were decreased in the peri-infarct area and were expressed in cardiac fibroblasts [140, 141]. Moreover, miR-144 knockout mice demonstrated impaired late remodeling after MI, which was reflected by elevated total cardiac collagen content [142]. Interestingly, intermittent aerobic exercise could enhance the expression of cardiac miR-101a in rats with MI, and miR-101a was associated with decreased expression of fibrotic genes, such as *Tgfb*, *fos*, *Smad2/3*, *Col1A1*, and *Col3A1* [143]. These findings indicate that cardiac miRNAs (miR-208, miR-101, miR-150, and miR-144) play a central role in fibrosis after MI. Overall, miRNAs may become a potential therapeutic target for myocardial fibrosis (miRNAs are briefly summarized in Table 2).

## 6.Conclusion

Myocardial fibrosis, as a main component of most cardiovascular diseases, has been a major focus in recent years. Endomyocardial biopsy, which is the gold standard for the diagnosis of myocardial fibrosis, has limitations in terms of clinical application, whereas biomarkers seem to be easier and safer in terms of diagnosis, therapeutic monitoring, and prognosis. With the development of technology, the investigation of myocardial fibrosis biomarkers has received attention in clinical and research communities. A systematic review of biomarkers and pathological effects of myocardial fibrosis is presented in Fig. 2.

When selecting biomarkers in experiments, researchers should consider the purpose and method of the experiment. Some biomarkers are very likely “bystander” markers, but many are functional factors that are closely related to collagen synthesis and degradation. Specifically, PICP, PINP, and PIIINP are suitable representatives of the mechanism of collagen synthesis in target organ injury in myocardial fibrosis. CITP, MMPs, and TIMPs reflect collagen degradation, and the balance of collagen synthesis and degradation in turn indicates the stability of organ fibrosis. Therefore, PICP, PINP, PIIINP, CITP, MMPs, and TIMPs are functional factors that can directly reflect the degree of fibrosis. Moreover, in the process of fibrosis, collagen metabolism is affected by many molecules, such as TGF-β, Smads, CTGF, corin, mesenchymal cell products, and inflammatory factors. CTGF induces proliferation of fibroblasts and increases extracellular matrix content. Corin affects heart function by regulating natriuretic peptides. Inflammation always accompanies fibrosis, and hence inflammatory markers can reflect the relationship between them. EndoMT is one of the important sources of fibroblasts, and TGF-β, Smads, and miRNA are the main regulators of collagen gene expression. Therefore, TGF-β, Smads, CTGF, corin, mesenchymal cell products, and inflammatory factors are “bystander” markers that can indirectly affect the fibrosis process.

It is important to note that fibrosis occurs not only in the heart, but also in other organs, so that changes in biomarker levels may not have only a cardiac origin [25]. In another respect, biomarkers must be strictly tested to determine whether they strongly reflect myocardial fibrosis. Endomyocardial biopsy can be used to estimate the usefulness and accuracy of biomarkers. Only when these initiatives are successful can biomarkers be incorporated into clinical practice. Additionally, cost-effectivity issues should also be taken into consideration, as the measurement of many biomarkers mentioned is not cheap, particularly when using a multiple-biomarker approach. Moreover, there is not currently a well-tested biomarker for fibrosis that is equivalent to NT-proBNP for HF. On the whole, the use of biomarkers is helpful in the assessment of myocardial fibrosis; therefore, more prospective studies are needed in the future.
